# Predictive value of gB2 antibodies for maternal–fetal transmission after primary cytomegalovirus infection treated with valacyclovir

**DOI:** 10.1002/uog.70206

**Published:** 2026-03-30

**Authors:** K. O. Kagan, A. L. Bissinger, O. Roth, J. Sonek, M. Hoopmann, Y. Ville, M. Enders

**Affiliations:** ^1^ Department of Obstetrics and Gynaecology University of Tübingen Tübingen Germany; ^2^ Institute of Medical Virology and Epidemiology of Viral Diseases University of Tübingen Tübingen Germany; ^3^ Fetal Medicine Foundation USA Dayton OH USA; ^4^ Division of Maternal Fetal Medicine Wright State University Dayton OH USA; ^5^ EA Fetus, Paris Descartes University, University of Paris Paris France; ^6^ Department of Obstetrics, Fetal Medicine and Surgery, Necker–Enfants Malades Hospital, AP–HP Paris France; ^7^ Laboratory Prof. Dr. G. Enders and Colleagues, MVZ Stuttgart Germany

**Keywords:** CMV, gb2 antibodies, pregnancy, transmission, valacyclovir

## Abstract

**Objective:**

To estimate the residual risk of maternal–fetal transmission after primary cytomegalovirus (CMV) infection in the periconceptional period or the first trimester in women treated with valacyclovir, and to assess whether the presence of glycoprotein B2 (gB2)‐specific immunoglobulin (Ig)‐G antibodies on immunoblot analysis refines fetal risk prediction.

**Methods:**

This was a retrospective single‐center study conducted at University Hospital Tübingen, Tübingen, Germany, between October 2023 and June 2025. Pregnant women with a primary CMV infection diagnosed < 14 weeks' gestation who received valacyclovir (8 g/day) were included in the study. The diagnosis of a recent primary CMV infection was based on maternal serology results, specifically positive anti‐CMV‐immunoglobulin (Ig)‐G and ‐IgM levels, and low or intermediate IgG avidity. Additionally, prior to initiation of valacyclovir treatment, the presence or absence of glycoprotein B (gB)2‐specific IgG antibodies was determined using a commercial line immunoblot. The primary outcome was maternal–fetal transmission of CMV detected at the time of second‐trimester amniocentesis.

**Results:**

Eighty‐five women met the inclusion criteria, including 47 with periconceptional and 38 with first‐trimester maternal CMV infection. Median maternal and gestational age at the time of diagnosis of primary maternal CMV infection were 33.1 (interquartile range (IQR), 29.9–34.9) years and 7.4 (IQR, 6.4–8.9) weeks, respectively. Amniocentesis was performed at a median gestational age of 20.6 (IQR, 20.1–21.0) weeks. Overall, maternal–fetal transmission was detected in 7/85 (8.2%) cases at the time of amniocentesis. All CMV transmissions occurred in women without gB2‐specific IgG antibodies (7/55 (12.7%)), while no transmissions occurred in those with gB2‐specific IgG antibodies (0/30 (0%)) (*P* = 0.048).

**Conclusions:**

In women with a primary CMV infection treated with valacyclovir, the presence of gB2‐specific IgG antibodies on immunoblot analysis identifies a subgroup with a low residual risk of maternal–fetal transmission. Incorporating gB2‐specific IgG antibodies status into risk stratification may improve patient counseling and clinical decision‐making. © 2026 The Author(s). *Ultrasound in Obstetrics & Gynecology* published by John Wiley & Sons Ltd on behalf of International Society of Ultrasound in Obstetrics and Gynecology.

## INTRODUCTION

Primary cytomegalovirus (CMV) infection remains the most important viral infection during pregnancy. In Western societies, in which approximately half of women are seropositive at the beginning of pregnancy, the primary infection rate is about 1–2%, and around 0.2–0.6% of newborns are infected^1^, ^2^, ^3^, ^4^, ^5^, ^6^. The fetal risk from a primary maternal CMV infection depends on the timing of maternal infection. The fetal transmission rate increases with gestational age, but the risk of developmental sequelae decreases. For primary maternal CMV infection in the pre‐ and periconceptional periods, the fetal transmission rates are 5.5% and 21.0%, respectively. In the first, second and third trimesters, the fetal transmission rates increase to 36.8%, 40.3% and 66.2%, respectively. The risk of long‐term developmental disabilities following a fetal CMV infection in the periconceptional period and in the first, second and third trimesters is 28.8%, 19.3%, 0.9% and 0.4%, respectively^7^.

If a maternal CMV infection occurs during the periconceptional period or in the first trimester, the main goal should be the prevention of maternal–fetal transmission. For this purpose, administration of valacyclovir is currently recommended by various expert guidelines^3^, ^6^. D'Antonio *et al*.^8^ summarized the results of three studies, which included 164 women treated with valacyclovir, and reported a fetal transmission rate of 7.5% and 18.7% in the periconceptional period and in the first trimester, respectively. Moreover, Chatzakis *et al*.^9^ performed an individual patient meta‐analysis using a generalized linear mixed model based on the same three studies. The authors found that the fetal transmission rates in the periconceptional period and in the first trimester were reduced by about 35% with valacyclovir treatment^9^.

One of the cornerstones of prevention using valacyclovir is to start treatment as soon as possible after the onset of the primary maternal infection. As the infection is often asymptomatic, the presence of both anti‐CMV‐immunoglobulin (Ig)‐G and ‐IgM antibodies with low or intermediate avidity is used to make the diagnosis of a recent infection. In addition to conventional serology, the onset of a primary infection can be further narrowed down using immunoblot analysis^10^, ^11^, ^12^, ^13^. These tests use phase‐specific and avidity antigens to determine the onset of an infection. Most importantly, in most patients, the glycoprotein B (gB) domain‐specific IgG response develops 6–8 weeks after the onset of infection^12^, ^14^, ^15^.

In this study, we assessed the residual risk of maternal–fetal transmission in pregnancies with a recent primary maternal CMV infection in the periconceptional period or the first trimester that were treated with valacyclovir. In addition to conventional CMV serology, we investigated the complementary use of gB2‐specific IgG antibody detection using immunoblot in fetal risk assessment.

## METHODS

This was an observational study conducted at the Department of Prenatal Medicine at the University Hospital Tübingen, Tübingen, Germany, between October 2023 and June 2025. Pregnant women were included in the retrospective analysis if a recent primary maternal CMV infection was diagnosed in the periconceptional period or in the first trimester. Only patients who started valacyclovir treatment before 14 weeks' gestation were included in the study.

Laboratory testing that led to the diagnosis of a primary maternal CMV infection was carried out either in the Institute of Medical Virology and Epidemiology of Viral Diseases at the University Hospital Tübingen or at the laboratory of Prof. Gisela Enders and colleagues in Stuttgart, Germany. Diagnosis of a recent primary CMV infection was based on the following maternal serology results: positive anti‐CMV‐IgG and ‐IgM levels, and low or intermediate IgG avidity. As different methods were used to determine IgG avidity, we did not use the absolute value but grouped the avidity into ‘low’ or ‘intermediate’ classes according to the manufacturer's specifications. In addition, a commercial CMV IgG immunoblot (immunoassay recomLine CMV IgG, IgM, IgG Avidity; Mikrogen, Neuried, Germany) was used prior to initiation of valacyclovir treatment to examine if domain‐specific IgG antibodies directed against a recombinant gB2 were present. According to the manufacturer, the presence of gB2‐specific IgG antibodies indicates that the infection occurred at least 6–8 weeks prior to the blood analysis. The results of the immunoblot were grouped according to the presence or absence of gB2‐specific IgG antibodies based on an assessment of the specific band intensity compared with a cut‐off control band, either by subjective visual reading or by automated reading using recomScan software (Mikrogen) for evaluation of recomLine strip tests.

A periconceptional infection was assumed if the blood sample indicating a recent CMV infection was taken prior to 8 + 0 weeks. For women from whom the blood sample was taken between 8 + 0 and 13 + 6 weeks, the time of infection was determined based on the IgG avidity. If the CMV‐IgG avidity was low or not measurable owing to low anti‐CMV‐IgG levels, the onset of infection was assumed to be in the first trimester, whereas if the CMV‐IgG avidity was intermediate, the onset of infection was assumed to be in the periconceptional period^16^. Pregnant women who met the inclusion criteria of periconceptional or first‐trimester primary CMV infection were offered treatment with valacyclovir. Prior to starting treatment, they were informed about the off‐label use of the drug, the associated risks and that the treatment complied with national and international guidelines^3^, ^17^.

All included women were offered amniocentesis, which was carried out at least 6 weeks after the diagnosis of primary CMV infection, at approximately 20 weeks. In the absence of documented maternal–fetal transmission, no further treatment was carried out. In the case of maternal–fetal transmission, continued treatment with 8 g/day of valacyclovir was offered as tertiary prophylaxis (off‐label use)^6^, ^17^. Further treatment options discussed included pregnancy termination, however none of the included women opted for termination of pregnancy as a management option.

Within a few days after delivery, neonates were tested for CMV in blood, urine and/or saliva using a nucleic acid amplification test and/or virus isolation in tissue culture. Information about the fetal development, transmission status at the time of amniocentesis and outcome data after birth were recorded in the perinatal databases of the center (Viewpoint, Solingen, Germany). The study was approved by the ethical committee of University Hospital Tübingen (approval number: 374/2025BO2).

### Virological studies

Depending on the initial findings, the following commercial tests were used at one of the two sites to clarify the CMV infection status and time of infection: Liaison CMV IgG, IgM and IgG avidity (DiaSorin, Saluggia, Italy); Elecsys CMV IgG, IgM and IgG avidity (Roche Diagnostics, Basel, Switzerland); Architect CMV IgG, IgM and IgG avidity (Abbott, Wiesbaden, Germany); recomLine CMV IgG, IgM and IgG avidity (Mikrogen); Vidas CMV IgG and IgG avidity (bioMérieux, Marcy‐l'Etoile, France); and SERION ELISA classic cytomegalovirus IgG and IgM (Virion/Serion, Würzburg, Germany).

The amniotic fluid was tested using two polymerase chain reaction assays (PCRs) (nested PCR (nPCR) and quantitative real‐time PCR), short‐term microculture and long‐term viral culture until generation of a cytopathic effect, over a period of 18 h to 10 days. Short‐term 18‐h fibroblast microculture, followed by CMV‐IE1 immunoperoxidase staining and long‐term (10 days) virus isolation from amniotic fluid, were performed using a virus‐concentration step with high‐speed centrifugation at 50000 *g* for 1 h at 4°C prior to virus inoculation. DNA was purified by spin columns using a QIAmp DNA Blood Mini Kit (Qiagen, Düsseldorf, Germany) and used for qualitative nPCR of the IE1Ex4‐target region. The limit of detection for nPCR was 200 copies/mL. Quantitative real‐time PCR from plasma, serum or EDTA whole blood, as well as from amniotic fluid, was performed using CMV‐R‐GENE® real‐time PCR kits (Argene®; bioMérieux) with a quantitative limit of detection of 500 copies of CMV‐DNA/mL. The assays were used as described in previous virological monitoring studies^18^.

### Statistical analysis

The risk of maternal–fetal transmission at the time of amniocentesis was examined according to the estimated time of maternal CMV infection (periconceptional *vs* first‐trimester infection) and according to the presence or absence of gB2‐specific IgG antibodies. Continuous variables are expressed as median (interquartile range (IQR)) and were compared using the Mann–Whitney *U*‐test. Categorical data are presented as *n* (%) and were analyzed using Fisher's exact test (two‐sided). For proportions, 95% CIs were calculated using the method of Clopper and Pearson. Univariable and multivariable logistic regression analyses were used to determine significant covariates for maternal–fetal transmission. To address sparse data and complete separation in categorical predictors, we applied ridge‐penalized logistic regression as an approximation to the Firth bias‐reduced estimator. The penalty strength was set to α = 0.01 to stabilize parameter estimates while retaining approximate maximum‐likelihood behavior. Two‐sided *P*‐values and 95% CIs were obtained via percentile bootstrap resampling (B = 1000). *P* < 0.05 was used to indicate statistical significance.

## RESULTS

**Figure 1:**
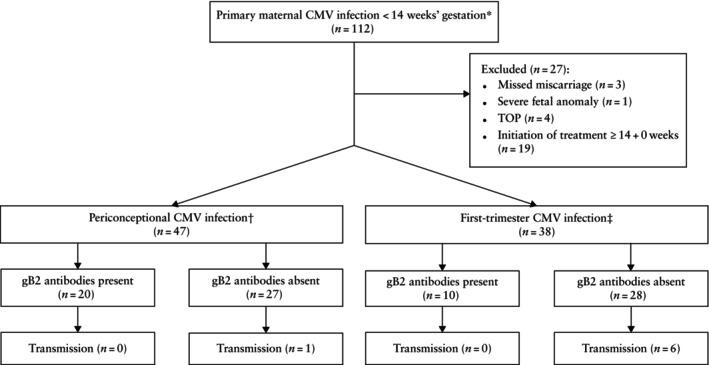
Flowchart summarizing cases of maternal–fetal transmission of cytomegalovirus (CMV) detected at the time of amniocentesis in women with recent primary CMV infection diagnosed before 14 weeks' gestation. 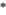
Primary infection was defined as positive anti‐CMV‐immunoglobulin (Ig)‐G and ‐IgM levels, and low or intermediate IgG avidity. †Periconceptional infection was assumed if blood sample indicating infection was obtained < 8 + 0 weeks, or between 8 + 0 and 13 + 6 weeks with intermediate IgG avidity. ‡First‐trimester infection was assumed if blood sample was obtained between 8 + 0 and 13 + 6 weeks with low IgG avidity. gB2, glycoprotein B2.

The database search identified 112 women who were referred to University Hospital Tübingen owing to a recent primary CMV infection before 14 weeks' gestation. Twenty‐seven women were excluded from further analysis owing to missed miscarriage (*n* = 3), severe fetal anomaly unrelated to CMV infection (*n* = 1), termination of pregnancy (*n* = 4) or treatment initiation ≥ 14 + 0 weeks (*n* = 19). Therefore, the study population comprised 85 women (Figure 1).

The median maternal age at the time of diagnosis of primary CMV infection was 33.1 (IQR, 29.9–34.9) years. Sixty‐six (77.6%) women had at least one previous child and in 60/66 (90.9%) cases, the youngest child in the family was 3 years of age or younger. The first blood sample that led to a referral was obtained at a median gestational age of 7.4 (IQR, 6.4–8.9) weeks. The median gestational age at the time of first referral to our unit was 9.1 (IQR, 8.3–11.6) weeks, thus, at a median of 13.0 (IQR, 8.0–20.0) days after the first blood sample was taken. At referral, the maternal antibody status was reassessed, the immunoblot analysis was performed and valacyclovir therapy was started. Amniocentesis was performed in 83 women at a median gestational age of 20.6 (IQR, 20.1–21.0) weeks. Two women declined invasive testing; in these cases, neonatal testing was negative for CMV. These pregnancies were therefore classified as CMV negative at amniocentesis for the purposes of the analysis. Further details about the study population and the treatment characteristics are presented in Table 1.

**Table 1:** Maternal and pregnancy characteristics of 85 women with primary cytomegalovirus (CMV) infection diagnosed < 14 weeks' gestation, according to maternal–fetal CMV transmission status at time of amniocentesis

Characteristic	No maternal–fetal transmission (*n* = 78)*	Maternal–fetal transmission (*n* = 7)	*P* †
Maternal age (years)	32.7 (29.9–34.9)	34.2 (31.7–35.3)	0.221
Gravidity	2 (2–3)	2 (2–2)	0.825
Parity	1 (1–1)	1 (1–1)	0.240
GA at blood sampling (weeks)	7.3 (6.4–8.6)	9.3 (7.3–10.9)	0.061
GA at initiation of valacyclovir (weeks)	9.3 (8.1–11.4)	11.7 (9.3–13.4)	0.050
Interval between diagnosis and initiation of therapy (days)	13.0 (8.0–18.0)	14.0 (9.0–21.0)	0.317
GA at amniocentesis (weeks)‡	20.6 (20.3–21.0)	20.9 (20.1–20.9)	0.754

Data are given as median (interquartile range).

* Two women declined invasive testing, but neonates were identified as CMV‐negative after birth, thus we included them in the group of women with no maternal–fetal CMV transmission at amniocentesis.

† Mann–Whitney *U*‐test.

‡ Data missing for two cases that did not undergo amniocentesis. GA, gestational age.

Overall, there were 47 periconceptional and 38 first‐trimester maternal CMV infections. At the time of amniocentesis, seven (8.2% (95% CI, 3.4–16.2%)) cases of maternal–fetal transmission were identified, of which six (15.8% (95% CI, 6.0–31.3%)) transmissions occurred within the first‐trimester‐infection group and one (2.1% (95% CI, 0–11.3%)) occurred within the periconceptional‐infection group. It is of note that, after birth, we observed a total of nine cases with a neonatal CMV infection.

At the time of diagnosis of the primary maternal CMV infection, gB2‐specific IgG antibodies were present in 30 (periconceptional group, *n* = 20; first‐trimester group, *n* = 10) subjects and absent in 55 (periconceptional group, *n* = 27; first‐trimester group, *n* = 28) subjects (Figure 1, Table 2). In total, all seven cases of maternal–fetal transmission detected at amniocentesis occurred in the group of 55 women without detected gB2‐specific IgG antibodies (12.7% (95% CI, 5.3–24.5%)), while no case of maternal–fetal transmission was identified among the 30 pregnancies with detected gB2‐specific IgG antibodies (0% (95% CI, 0–11.6%)) (*P* = 0.048). In the group with periconceptional primary maternal CMV infection, the transmission rate was similar when stratified by the presence or absence of gB2‐specific IgG antibodies (0% (0/20) *vs* 3.7% (1/27), respectively). However, in the group with first‐trimester CMV infection, the transmission rate was 0% (0/10) when gB2‐specific IgG antibodies were present and 21.4% (6/28) when absent (Figure 1).

**Table 2:** Distribution of pregnancies with and those without maternal–fetal transmission of cytomegalovirus (CMV) according to presence or absence of glycoprotein B2 (gB2)‐specific immunoglobulin G (IgG) antibodies, IgG avidity status and time of primary maternal CMV infection

	gB2‐specific IgG absent		gB2‐specific IgG present	
Time of CMV infection/parameter	No Transmission (*n* = 48)	Transmission (*n* = 7)	No Transmission (*n* = 30)	Transmission (*n* = 0)
Periconceptional	26 (54.2)	1 (14.3)	20 (66.7)	0 (0)
Low IgG avidity	11/26 (42.3)	0/1 (0)	3/20 (15.0)	0/0 (0)
Intermediate IgG avidity	15/26 (57.7)	1/1 (100)	17/20 (85.0)	0/0 (0)
First‐trimester	22 (45.8)	6 (85.7)	10 (33.3)	0 (0)
Low IgG avidity	22/22 (100)	6/6 (100)	10/10 (100)	0/0 (0)
Intermediate IgG avidity	0/22 (0)	0/6 (0)	0/10 (0)	0/0 (0)

Data are given as *n* (%) or *n*/*N* (%).

Logistic regression analysis was performed to identify maternal, pregnancy and infection parameters that were significantly associated with maternal–fetal transmission at the time of amniocentesis in the second trimester. On univariable logistic regression analysis, we observed a significant association between reduced odds of maternal–fetal transmission rate and the presence of gB2‐specific IgG antibodies, as well as with gestational age at blood sampling and gestational age at initiation of valacyclovir therapy. On multivariable logistic regression analysis, only the presence of gB2‐specific IgG antibodies significantly reduced the odds of maternal–fetal CMV transmission (Table 3).

**Table 3:** Logistic regression analysis to identify covariates associated with maternal–fetal cytomegalovirus transmission detected at the time of amniocentesis

	Univariable		Multivariable	
Covariate	OR (95% CI)	*P*	aOR (95% CI)	*P*
Maternal age (in years)	1.091 (0.906–1.313)	0.358	—	—
GA at blood sampling (in weeks)	1.501 (1.011–2.228)	0.044	1.074 (0.415–2.202)*	0.916
GA at initiation of valacyclovir therapy (in weeks)	1.588 (1.038–2.430)	0.033	1.455 (0.855–4.628)*	0.550
Interval between diagnosis and initiation of therapy (in days)	1.016 (0.942–1.096)	0.679	—	—
GA at amniocentesis (in weeks)	1.082 (0.681–1.719)	0.738	—	—
Intermediate IgG avidity	0.240 (0.028–2.087)	0.196	—	—
gB2 antibodies present	0.002 (0.001–0.007)*	0.004	0.002 (0.000–0.009)*	0.004

* Penalized logistic regression (ridge, α = 0.01) used as Firth approximation to mitigate separation; 95% CIs and two‐sided *P*‐values derived from percentile bootstrap (B = 1000). aOR, adjusted odds ratio; GA, gestational age; gB2, glycoprotein B2; IgG, immunoglobulin G; OR, odds ratio.

## DISCUSSION

In this study, we found that the residual risk of maternal–fetal CMV transmission at the time of amniocentesis performed at approximately 20 weeks, following prophylactic therapy with valacyclovir initiated before 14 weeks, was 8%. Furthermore, the residual risk of transmission was significantly lower if gB2‐specific IgG antibodies were present at the time of diagnosis of maternal primary infection (0% *vs* 12.7%; *P* = 0.048), with significantly reduced odds of transmission (adjusted odds ratio, 0.002 (95% CI, 0.000–0.009); *P* = 0.004). The difference in the risk of maternal–fetal transmission was also pronounced in pregnancies in which CMV infection occurred in the first trimester (15.8%) compared with infection in the periconceptional period (2.1%).

Three studies have examined the effect of valacyclovir on the maternal–fetal transmission rate following a periconceptional or first‐trimester primary maternal CMV infection. The results were summarized by Chatzakis *et al*.^9^, who reported that valacyclovir reduced the maternal–fetal transmission rate in women with a periconceptional or first‐trimester CMV infection. The overall transmission rates were 11.1% and 25.5% in the treatment and placebo groups, respectively. In the periconceptional infection group, the authors observed a maternal–fetal transmission rate of 6.5% in women treated with valacyclovir and 14.6% in those without treatment. In those with a first‐trimester infection, the respective rates of transmission were 17.0% and 36.7%. In our study, in which all subjects were treated with valacyclovir, the transmission rate in the periconceptional group was only 2.1% but the 95% CIs include the results of those previous studies. In the group with first‐trimester infection, we observed a maternal–fetal transmission rate of 15.8%, which is also in line with the previous studies. However, we acknowledge that the lower transmission rate in our study could be a consequence of the fact that we included women with low or intermediate IgG avidity.

Interestingly, women with gB2‐specific IgG antibodies had a significantly lower transmission rate than did women without gB2‐specific IgG antibodies. This is particularly important for women with a first‐trimester infection, for whom the residual risk of maternal–fetal transmission was 0% *vs* 21.4% in those with *vs* without gB2‐specific antibodies, respectively. One potential explanation for this finding is that it generally takes several weeks after the onset of a primary CMV infection before gB2‐specific IgG antibodies can be detected^13^. As such, suspected cases of first‐trimester infection with gB2‐specific IgG antibodies detected may in fact be cases in which infection occurred during the periconceptional period, a time with a much lower risk of maternal–fetal transmission. By the same reasoning, it is possible that some women included in the periconceptional‐infection group may instead have had a preconceptional infection, which is also associated with a lower *a‐priori* risk of maternal–fetal transmission.

Immunoblot analysis uses recombinant phase‐specific and avidity antigens, in particular immunogenic domains from non‐structural (e.g. pp68‐72 (IE‐1), pp52 (CM2)) and structural (e.g. gp58/116 (gB), tegument phosphoprotein pp150, capsid protein pp28) CMV proteins, to determine the onset of an infection. IgG antibodies to the major DNA binding protein (pp52) have been shown to be present mainly in early primary CMV infection, whereas IgG antibodies to the major tegument phosphoprotein (pp150) predominate during the convalescence phase and thereafter^19^. Immunoblot avidity determination is generally in good agreement with conventional avidity testing^11^. In contrast to conventional immunoassays, the immunoblot enables further analysis of the immune response towards individual antigens (e.g. gB2). Several studies have described a delay in the appearance of gB2‐specific IgG antibodies in the course of primary CMV infection. Schoppel *et al*.^13^ found a delay of 50–100 days in the appearance of gB‐specific antibodies in pregnant women with a primary CMV infection. A minimum lag period of 60 days between infection and the detection of gB‐specific IgG antibodies has also been reported in transplant patients^14^. Therefore, detection of gB‐specific IgG antibodies can supplement conventional testing, especially in the presence of equivocal serology results^10^, ^14^, ^15^.

The idea of protective gB2‐specific IgG antibodies was the rationale behind the treatment of CMV with hyperimmunoglobulins^16^. However, in contrast to our previous results^16^, a more recent prospective study conducted by our group did not show a positive effect of immunoglobulin treatment on the maternal–fetal transmission rate^20^. We can only speculate why the beneficial effect seen in previous studies was not replicated in this later study^20^. For example, perhaps the presence of gB2‐specific IgG antibodies is not protective itself, but is a surrogate marker for an older infection with a lower maternal–fetal transmission rate due to a more complex immune response than the absence or presence of a specific type of antibody.

There are some limitations of the present study to consider. Mainly, this was a retrospective single‐center study and it was therefore prone to potential selection bias. Furthermore, there was heterogeneity in the commercial tests used. Therefore, IgG avidity could not be analyzed as a continuous variable and was instead treated as a categorical variable. Finally, the study was not designed to evaluate an important aspect of valacyclovir treatment, namely maternal complications.

In conclusion, we have shown that the presence of gB2‐specific IgG antibodies can be helpful in predicting the residual risk of maternal–fetal transmission in women with a periconceptional or first‐trimester primary CMV infection who are treated with valacyclovir. In our study, we did not observe any case of maternal–fetal transmission in women with gB2‐specific IgG antibodies, while the transmission rate was almost 13% in women without gB2‐specific IgG antibodies.

## Data Availability

The data that support the findings of this study are available on request from the corresponding author. The data are not publicly available due to privacy or ethical restrictions.
